# Functional recovery of a subtropical evergreen-deciduous broadleaved mixed forest following clear cutting in central China

**DOI:** 10.1038/s41598-018-34896-5

**Published:** 2018-11-07

**Authors:** Yongtao Huang, Xiao Zhang, Runguo Zang, Shenglei Fu, Xunru Ai, Lan Yao, Yi Ding, Jihong Huang, Xinghui Lu

**Affiliations:** 1 Post-Doctoral Research Program of Geography, College of Environment and Planning, Henan University, Kaifeng, 475004 PR China; 2 Key Laboratory of Forest Ecology and the Environment, the State Forestry Administration, Institute of Forest Ecology, Environment and Protection, Chinese Academy of Forestry, Beijing, 100091 PR China; 3 College of Environment and Planning, Henan University, Kaifeng, 475004 PR China; 4 Co-Innovation Center for Sustainable Forestry in Southern China, Nanjing Forestry University, Nanjing, Jiangsu 210000 PR China; 5 School of Forestry and Horticulture, Hubei University for Nationalities, Enshi, Hubei 445000 PR China

## Abstract

Ecosystem functioning is largely dependent on the functional traits of its component species. Most of the previous researches on ecosystem recovery have mainly focused on taxonomic composition but less attention is concentrated on functional community composition. Here, we examine the dynamic trend of functional community composition along a recovery chronosequence following clear cutting in subtropical evergreen-deciduous broadleaved mixed forest. Results showed that with the process of recovery, the functional composition changed from a community with high specific leaf area (CWM_ SLA), leaf nitrogen concentration (CWM_ LNC) and leaf phosphorus (CWM_ LPC) but low leaf thickness (CWM_ LT) and stem tissue density (CWM_ STD) to that with low CWM_ SLA, CWM_ LNC and CWM_ LPC but high CWM_ LT and CWM_ STD. Functional traits of evergreen and deciduous species were significantly different in each stage. Light availability and soil phosphorus were the most important influencing factors during the recovery. Our study suggests that the subtropical evergreen-deciduous broadleaved mixed forest is gradually shifting from a resource acquisitive to a resource conservative assemblage, in which evergreen species will become more and more dominant. Any management or conservation planning upon the forest ecosystem should integrate this dynamic trend of functional change.

## Introduction

Disturbance is one of the most important forces in structuring biotic communities^1^. The majority of the earth’s forests are secondary growth due to varied forms of disturbances^2^. Forest recovery after disturbance is a process of gradual change in species composition and community composition over time^3^. Processes shaping the patterns of forest succession have traditionally been approached by quantifying the changes in plant species composition over time^4–6^, yet this kind of research is often not closely relevant to understanding forest ecosystem functions^7^. Plant functional traits have proven to be an effective approach in analyzing changes in the structure and function of plant communities after disturbance^8^. Plant functional traits are the morphological, physiological, phenological features which affect plant fitness, and can represent species’ ecological strategies and determine how they respond to changing conditions, which might have effects on species in higher trophic levels and the functioning of the whole ecosystem^9^. Examining changes in the composition of functional traits in plant communities during recovery could help to better understand the underlying processes of succession^10^. Many functional trait researches have been conducted in individual species level^11^. However, community-weighted means of trait values (CWM) can largely determine how individual plant species contribute to ecosystem processes at the community level^12^. Community weighted mean trait values therefore have been widely adopted in ecological researches for summarizing different aspects of community trait composition^13^.

Forest recovery are characterized by important shifts in resources conditions (e.g. from high to low light availability), and species composition (e.g., from shade-intolerant to shade-tolerant species)^14^. An acquisitive-conservative continuum in plant resource-use strategies exists during succession, along which a species’ position can be determined by its functional traits^15,16^. The trade-off between acquisition and conservation strategies runs from fast-growing species with acquisitive resource-use strategies (rapid acquisition of resources, cheap-to-construct acquisitive leaves with high photosynthetic rates that maximize resource capture, and potential for quick returns on investments of nutrients, i.e. high specific leaf area and high leaf nutrients), to species with conservative resource-use strategies (invest less in growth, build leaves with traits leading to a more expensive-to-construct conservative leaves that tolerate stress and physical damage, and better conserve the acquired resources, i.e. low specific leaf area, sclerophyllous leaves, and low leaf nutrients)^15^. These trade-offs can be used to explain species’ successional perfectly. Species with functional traits that can exploit rich resources usually dominate in early successional stages while those species with conservative resource-use traits often dominate in late successional stages^2,17^. As succession proceeds, acquisitive resource strategy species are progressively replaced by long-lived, shade-tolerant resource conservative species^13,18^.

Studies on successional change from acquisitive to conservative strategies have been conducted in many forest types, where species replacement during forest succession is often explained by their adaptations to changing environments^19^. Different resource supply capacity under different environmental condition is thought to be one of the main forces driving plant functional traits changes during forest succession^20^. For example, fast-growing species with efficient resource acquisition traits often thrive in the light rich environment of early-successional stages, while slow-growing species with resource conservative traits dominate in the poor light conditions of late-successional stages^21^.

Changes in ecological strategies during secondary succession can be presumed to reflect transitions between deciduous and evergreen species. Deciduous and evergreen species can have fundamentally different physiology, which can be reflected in plant trait syndromes^22^. Deciduous species have commonly short-lived leaves while evergreens have coriaceous leaves with greater longevity^23^. Previous research has found that deciduous woody species show higher relative growth rates, higher SLA, and higher photosynthetic rates than evergreen species^24–26^. Compared with evergreen species, deciduous species have a shorter time each year to assimilate resources and generally require high nutrient conditions for efficient photosynthesis^27^.

Evergreen broad-leaved forests are abundant in the subtropical region of China and represent a globally distinct ecosystem between 25 and 35°N. With increasing altitude and decreasing temperatures, subtropical evergreen-deciduous broadleaved mixed forests become the dominant vegetation type in subtropical China^28^. The functional community composition of the subtropical evergreen-deciduous broadleaved has not been explored. The objective of this study is to examine changes in plant functional traits and the overall strategies along a natural recovery gradient represented by a chronosequence (20 years old second growth, 35 years old second growth and old growth) following clear cutting in a subtropical evergreen-deciduous broadleaved mixed forest in central China. Specifically, we ask the following questions: 1) How does functional community composition and ecological strategies change during recovery following clear cutting? 2) What are the differences in the average functional traits between deciduous and evergreen species during recovery? 3) Which environmental factors would lead to differences in functional composition among recovery stages?

## Results

### Changes in plant functional traits during recovery following clear cutting

**Figure 1:**
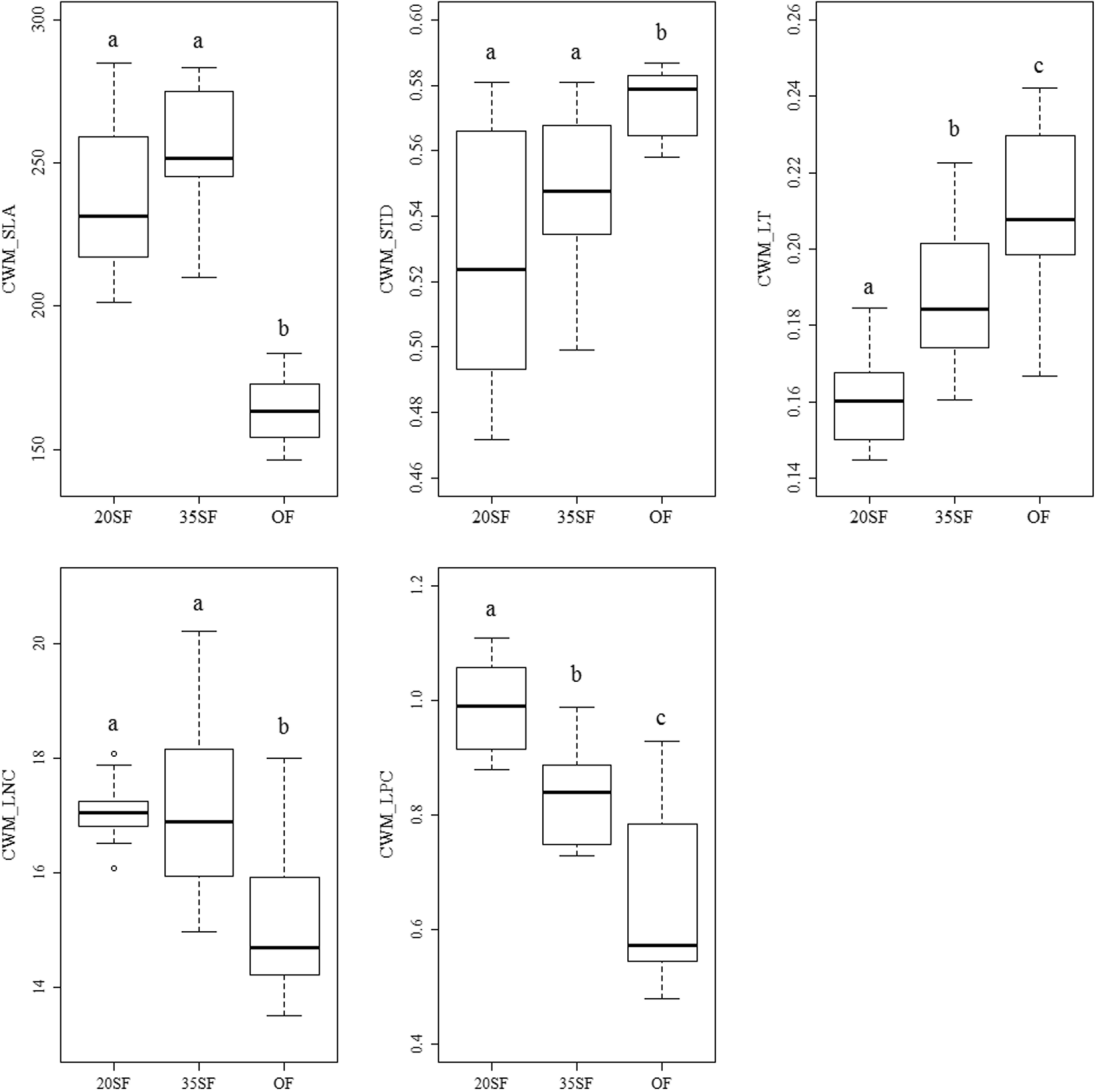
Variations of community-level plant functional traits in different recovery stages of the subtropical evergreen-deciduous broadleaved mixed forest after clear cutting. CWM_SLA, specific leaf area; CWM_LT, leaf thickness; CWM_STD, stem tissue density; CWM_LNC, leaf nitrogen concentration per mass; CWM_LPC, leaf phosphorus concentration per mass. 20SF, 20-year-old second growth forest; 35SF, 35-year-old second growth forest; OF, old growth forest. Data with different letters are significantly different (p < 0.05).

The five community level plant functional traits all changed significantly among different recovery stages (Fig. 1). The value of CWM_SLA in the old growth was significantly lower than those in the 20 and 35-year-old second growth (p < 0.01). CWM_SLA initially increased and then decreased with recovery. CWM_STD increased gradually with recovery though there was no significant difference in CWM_STD between the 20- and 35-year-old second growth (p > 0.05), but both of the secondary stages were significantly lower than that of the old growth. CWM_LT increased significantly with recovery (p < 0.01). CWM_LNC and CWM_LPC decreased gradually with recovery (p < 0.01). CWM_LNC between 20 and 35-year-old second growth was not significantly different (p > 0.05). Three patterns existed in the changes of community level plant functional traits, i.e. increased and then decreased (CWM_SLA), gradually increasing (CWM_STD, CWM_LT), and gradually decreasing (CWM_LNC, CWM_LPC) (Fig. 1).

**Figure 2:**
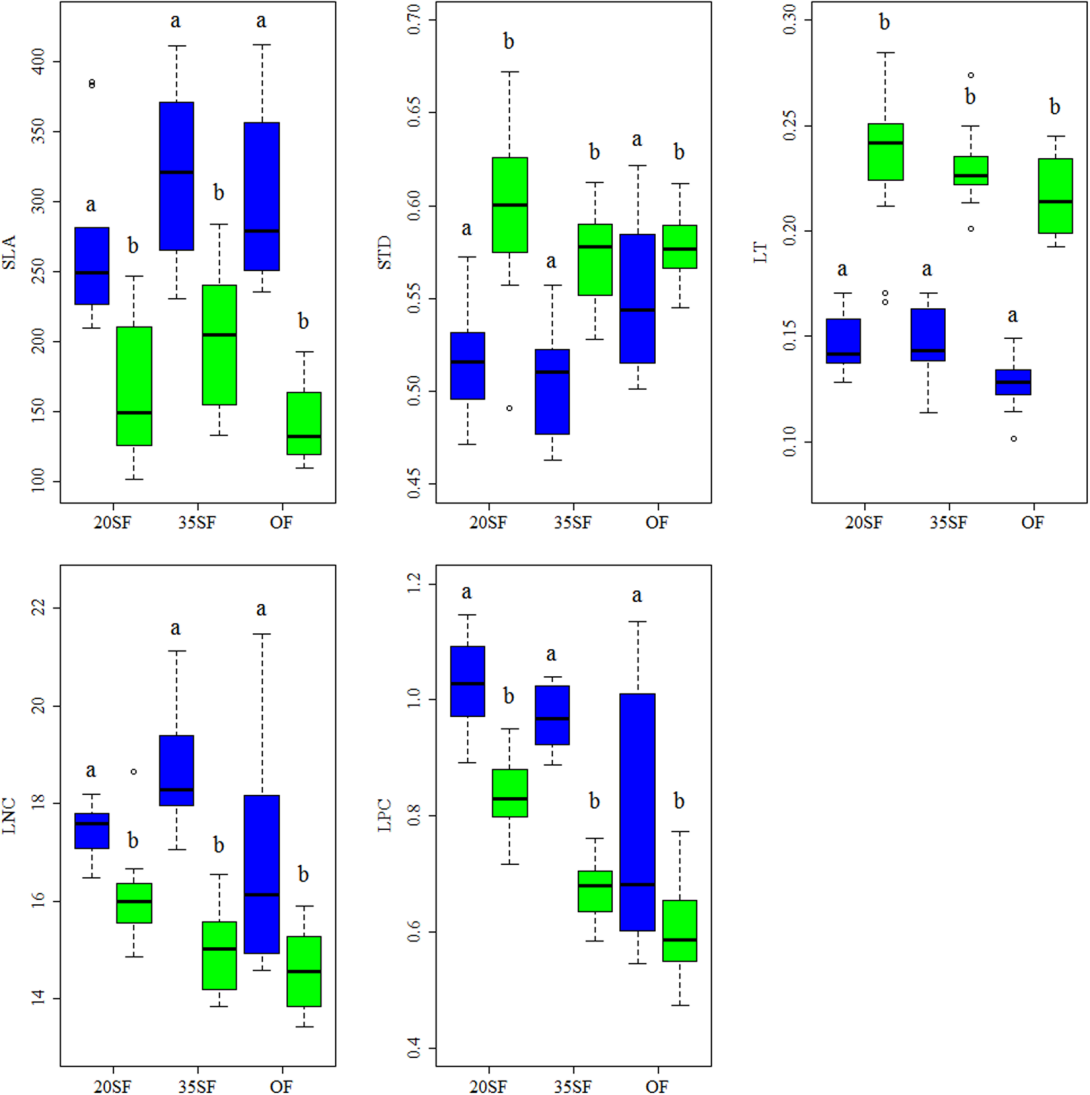
Plant functional traits (average value) for deciduous (blue) and evergreen (green) species during different recovery stages following clear cutting. Data with different letters are significantly different between deciduous and evergreen species (p < 0.05). Legends are as in Fig. 1.

In addition, all the average values of five measured traits differed significantly between deciduous and evergreen species in each recovery stage (p < 0.01) (Fig. 2). Evergreen species had higher LT and STD but lower SLA, LPC, and LNC than deciduous species in each recovery stage.

### Relationships between community level plant functional traits and environmental factors during recovery following clear cutting

Differences in environmental factors were found between different recovery stages (Table 1). Generally, SWC, SOM and the soil nutrients (TN, TP, AN and AP) decreased and then increased with recovery. Soil pH did not show any significant changes during the recovery. Canopy openness (CO) in the 20-year-old second growth was significantly higher than those in the 35-year-old second growth and old growth (p < 0.05).

**Table 1:** Environmental variables (mean ± SD) in the three recovery stages. Means with different letters are significantly different (p < 0.05).

Recovery stage	Environmental variables							
	SWC	pH	SOM	TN	AN	TP	AP	CO
	(%)		(g/kg)	(g/kg)	(mg/kg)	(g/kg)	(mg/kg)	(%)
SF20	0.38 ± 0.1b	4.67 ± 0.1a	86.2 ± 15b	6.17 ± 1.2ab	359.1 ± 68.5a	0.59 ± 0.1ab	0.17 + 0.04a	15.1 ± 1.7b
SF35	0.33 ± 0.1a	4.68 ± 0.3a	69.0 ± 18a	5.2 ± 0.9b	308.6 ± 63.3a	0.47 ± 0.1b	0.14 + 0.02a	12.77 ± 1.9a
OG	0.41 ± 0.1ab	4.65 ± 0.2a	81.2 ± 17ab	6.86 ± 2.6a	365.3 ± 98.0a	0.67 ± 0.3a	0.16 + 0.03a	10.27 ± 1.3a

**Figure 3:**
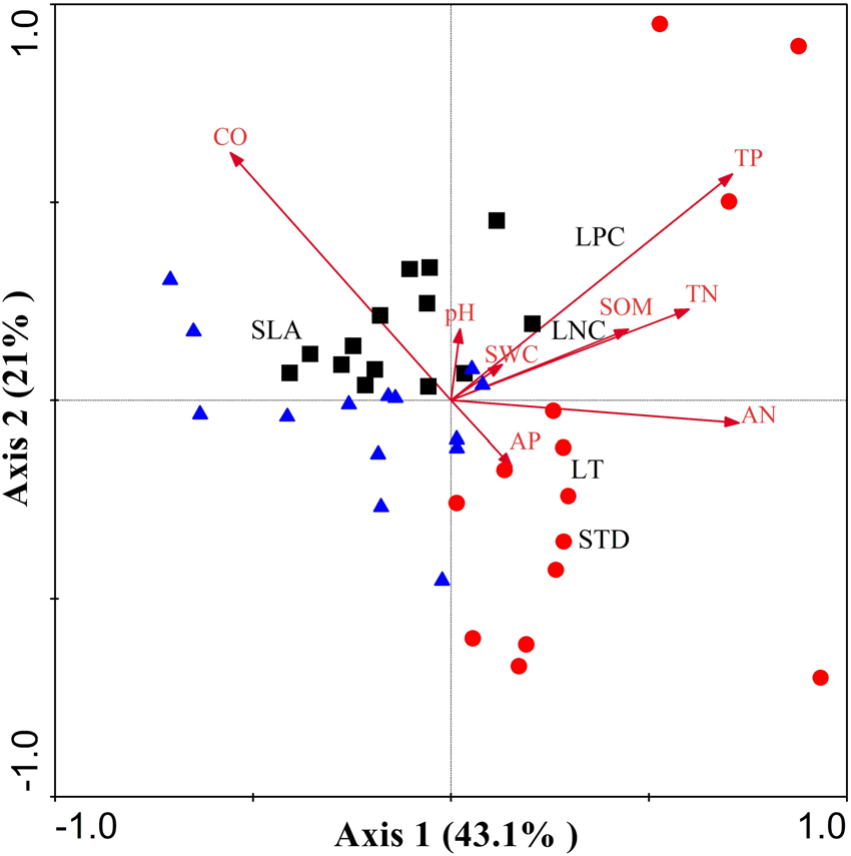
Redundancy analysis (RDA) ordination showing the relationship between the environmental variables and plant functional traits in the subtropical evergreen-deciduous broadleaved mixed forest of different recovery stages. Black square: 20-year-old second growth forest; blue triangle: 35-year-old second growth forest; red cycle: old growth forest; red arrow: soil properties; dark word: plant functional traits.

The RDA ordination analysis showed that correlations between community level plant functional traits and the measured environmental factors varied with different recovery stages (Fig. 3). All the measured parameters formed a clear gradient along the chronosequence. The first two axes explained 64.1% of the variation (43.1% and 21%). CO and TP were significantly explained the patterns of community composition (p < 0.05).

## Discussion

Plant functional traits have been recognized as a useful tool to predict plant responses to disturbances^29^. Our study showed that at community level, plants with high CWM_SLA, CWM_LNC and CWM_LPC but low CWM_LT and CWM_STD in earlier stages of recovery were progressively replaced by those with low CWM_SLA, CWM_LNC and CWM_LPC but high CWM_LT and CWM_STD (Fig. 1). The changes in functional traits were consistent with our previous studies on species diversity, that is, the richness and dominance of deciduous species gradually declined, and the evergreen species increased gradually in the community^28^. Those results suggest that secondary succession after clear-cutting not only changes species composition but also functional composition. Functional composition should be taken into account when studying subtropical secondary forests.

Species turnover may plays an important role in determining the directional shifts in functional traits along the recovery process (Supplementary Table S1). We found a decrease in deciduous species and increase of evergreen species as recovery progressed (Supplementary Fig. S1). In the early recovery stage (20 years), sites were dominated by pioneer species (including *Carpinus fargesiana*, *Cyclobalanopsis glauca*, *Weigela japonica* and *Dendrobenthamia japonica*), most of which were deciduous species. Deciduous species have higher SLA, LNC and LPC but lower LT and STD than evergreen species, suggesting that deciduous species have an overall high efficient resource use functional syndrome than the evergreen species^2,13^. During recovery, shade tolerant evergreen species increased while most of the early or mid-successional deciduous species were shaded out. Finally, the old growth forest was dominated by shade-tolerant climax evergreen species or a few long-lived pioneer deciduous, which had low SLA, LNC and LPC but high LT and STD^30^.

Our study on the variations in functional traits of subtropical evergreen-deciduous broadleaved mixed forest species suggest a shift of plant ecological strategies from resource acquisition(represented by deciduous species) to resource conservation(represented by evergreen species) during the recovery process, consistent with results from previous studies in other forests^15,31^. This kind of plant functional changes from an acquisitive to a conservative strategy suggests that leaf economic spectrums^16^ play an important role in functional community composition change during succession of the subtropical evergreen-deciduous broadleaved forest.

The difference between evergreen and deciduous species functional traits reflected a trade-off. Plant functional trait usually has a trade-off between growth and longevity^11^. Deciduous species with high SLA and leaf nutrients (such as LNC and LPC) often associated with an efficient light foraging^30^, high photosynthetic capacity and short leaf turnover time^32,33^. Deciduous trees have a limited time each year to photosynthesize and require high nitrogen for photosynthesis^27^, their thinner LT also can make gas exchange more efficient. Deciduous species also have a low STD with cheap volumetric construction costs in order to outcompete their neighbors. These traits facilitate the rapid growth of deciduous species and dominate the community in early recovery stages. Evergreen species with a low SLA, high LT and STD show relatively high construction costs^34^. This may result in longer leaf longevity of evergreen species, as more time is necessary to compensate the construction costs of the leaf. Those high construction costs may help them to withstand frost in low temperature or drought during stressful periods^35^. Additionally, leaves with lower SLA have more structural carbohydrates for support and likely fewer living tissues^36^, phosphorus in leaves may be more “diluted”, leading to an overall lower leaf nutrients of evergreen species^37^. All in all, leaves of deciduous species have functional syndroms implying a low construction cost, whereas the contrasting functional syndroms of the evergreen species suggesting a high construction cost^23^. Deciduous trees acquire available resources rapidly and have high growth rates but use tissues that are prone to damage. The opposite occurs with the slow growth evergreen trees having long-lived tissues that conserve and reuse internal resources more efficiently and keep growth for longer times^8,37^.

The results of this study clearly suggest that the distributions of functional composition values were mainly shaped by available light and soil phosphorus nutrient following recovery (Fig. 3). Light availability is a constraining factor for tree growth^38^, species evolved different functional traits to cope with the changing light conditions^39,40^. High canopy openness in the early stages created an ideal environment for light-demanding deciduous species. Owing to higher light availability in the early successional forests, pioneer deciduous species can acclimate better to high light conditions to realize high light-saturated photosynthetic rates, resulting in high growth rates. This in turn leads to high SLA, LNC, LPC, and low LT and STD in 20 and 35-year-old second growth forests. As recovery progresses, CO or light availability became lower and shade-tolerant evergreen species invaded the understory of the pioneer trees.

In many tropical and subtropical areas, soil P was found to be an important limiting nutrient for plant growth^41–43^. Our results corroborate those findings and indicate that soil P is the most important soil environmental factor affecting functional traits. It has been reported frequently that low soil N and soil P were usually associated with low LNC and LPC in plant leaves^36,44^. However, the availability of soil nutrients to plants may varied with the actual conditions of plant communities and abiotic environment^45,46^. Although relatively high soil nutrients were found in old growth forest, plants in old growth forest plots had lower LNC and LPC in our study area. This may be because evergreen plants allocate more nitrogen to cell walls proteins, which necessarily decreases their photosynthetic rates, leading to a reduction in the amount of photosynthetic proteins, resulting in the slow growth rates^47,48^.

## Conclusions

The trait-based approach has been proved to be a powerful way of examining ecosystem dynamics. Our results suggest that there were significant and consistent shifts in functional features of plant communities along the secondary successional gradient represented by the three stages of chronosequence following clear cutting. With the progress of natural recovery, the subtropical evergreen-deciduous broadleaved mixed forest is gradually shifting from a resource acquisitive to a more and more resource conservative assemblage, in which evergreen species will become functionally more and more important than deciduous species. Furthermore, the variation in functional composition of plant assemblage is in accordance with the changing abiotic conditions including light and soil P. Changes in functional compositions caused by clear cutting will need to be taken into account for future management.

## Materials and Methods

### Study site and field sampling

The study area is located in the 208 km^2^ Mulinzi National Nature Reserve (29°55′–30°10′N, 109°59′–110°17′E) in south-west Hubei Province, central China (Fig. S2). Elevation ranges from 1100 to 2095.6 m asl. The climate is humid subtropical monsoon with mean annual precipitation of 1733 mm, the majority of which falls from June to September. The mean annual temperature is 15.5 °C, with a mean monthly maximum of 26 °C in July and a mean monthly minimum of 4.6 °C in January. The dominant vegetation type is the subtropical evergreen-deciduous broadleaved mixed forest. Before the late 1970s most of the forests were subject to clear cutting, after which they were left to regenerate naturally as secondary forests.

Two recovery stages of 20 years old (SF20) and 35 years old (SF35) secondary forest since clear cutting were selected and compared to an old-growth forest (OF) in the study area. The 20 years old secondary forest has been undisturbed for 20 years and the 35 years old secondary forest has been undisturbed for 35 years. The old-growth forest has experienced no apparent anthropogenic.

Hereby, we refer these three successional stages of vegetation as the early, middle and late recovery stages of the studied forest. A total of 42 permanent plots of 20 m × 20 m were established with 14 in each recovery stage. Each of the 14 plots was randomly selected and was established as permanent forest dynamics plot (FDP) according to the standard protocols of CTFS (Center for Tropical Forest Science)^49^. In each plot, each woody stem (including trees, shrubs and lianas) with DBH (diameter at breast height) ≥1 cm was tagged and mapped, identified to the species, and DBH was recorded, leaf habits (deciduous or evergreen) of the species were confirmed according to the Flora of China (English edition; http://www.efloras.org).

### Measurement of environmental factors

Surface (0–20 cm depth) soil samples were collected from five randomly selected points in each plot and mixed into one sample. Soil samples were air-dried and sieved through 2 mm mesh. Soil water content (SWC), soil pH and soil nutrients were analyzed in the laboratory. Soil pH was measured using a 1:2.5 soil/water mixture and a digital pH meter. Soil available nitrogen (AN) was determined by the cornfield method, soil total nitrogen (TN) was determined by the semimicroKjeldahl method, soil organic matter (SOM) was determined by the K_2_Cr_2_O_7_ titration method after digestion, soil total phosphorus (TP) was determined colorimetrically after wet digestion with H_2_SO_4_ plus HClO4, soil available phosphorus (AP) was extracted with 3% (NH_4_)_2_CO_3_ solution. A hemispherical digital photograph was taken at 1.5 m above ground in the center of each plot by using a fish lens (SIGMA 8 mm F3.5 EX DG fisheye and Canon 450D digital camera). Canopy openness (CO) refers to the relative amount of sky that is visible from a point beneath the forest canopy and was calculated using the Gap Light Analyzer software^28^.

### Functional trait measurement

Five functional traits of all the 151 woody species were measured. For species with fewer than five individuals per plot, all individuals were sampled. For species with more than five individuals per plot, 5–10 well-developed individuals were sampled. For each individual, three recently expanded leaves were collected for further measurements. Leaf area was measured with a leaf area meter (LI-COR 3100 C Area Meter, LI-COR, USA). Specific leaf area (SLA, cm^2^ g^−1^) was calculated as leaf area divided by leaf dry mass (after drying for 72 h at 70 °C). Leaf nutrients characteristics, including leaf nitrogen concentration per mass (LNC, mg g^−1^) and leaf phosphorus concentration per mass (LPC, mg g^−1^) were measured using standardized protocols^4^. Leaf thickness (LT, mm) was measured as the average of three measurement of each lamina using a digital micrometer. To characterize species stem tissue density (STD, g cm^−3^), one 10-cm long segment was cut from branches between 1 and 2 cm in diameter. We removed the pith, phloem, and bark, and then measured fresh volume using a Mettler-Toledo balance. Density components as well as dry mass after drying for 72 h at 70 °C were also measured. We calculated stem tissue density as the dry mass divided by fresh volume based on the entire sectional area no matter the stem was hollow or not^9,50^.

### Statistical analysis

We coupled the species-trait matrix to the plot-species matrix to construct a plot trait matrix, and calculated community weighted mean (CWM) trait values. The CWM value is the proportional trait value by species abundance^51^. These calculations were based on the FD_var_ index using the FD package in R with the function dbFD^52^. It is calculated as follows:

1 $${\rm{CWM}}=\sum _{i=1}^{s}\,{W}_{i}\times {X}_{i}$$

where S is the total number of species, W_i_ is the relative abundance of the i^th^ species and X_i_ is the trait value of the i^th^ species. Plant functional traits of evergreen and deciduous trees were calculated as mean values.

Differences in functional traits among the three recovery stages were assessed by one-way analysis of variance. If variation was significant (p < 0.05), a Tukey honest significant difference multiple comparisons was used to determine where the significance differences lay. Step-wise multiple regression analysis between functional trait values and evergreen and deciduous species abundance in different recovery stages was used to validate their relationship.

Redundancy analysis (RDA) ordination was performed by relating the constructed site × trait matrices to the environmental factors matrix. We applied envfit function of the vegan packages^53^ to see which environmental factors were related to the correlation-based vectors of trait composition among sites (a random permutation test of 999 runs). Slope and elevation of each plot was measured, and mantel test was used to determine that they had no significant effect on plant functional traits (p > 0.05). All the statistical analyses were performed with R 3.1.3 Program.

### Ethical approval and informed consent

All applicable international, national, and/or institutional guidelines for the care and use of plants were followed. No ethical approval was required from an institutional or national ethics review board.

### Third party rights

All co-authors have read and agreed with the contents of the manuscript. We certify that the submission is an original work and is not under review at any other publication. We own intellectual property rights on our data. We agree to publish our manuscript in Scientific Reports (Nature Publishing Group, a division of Macmillan Publishers Ltd), to publish the image under an Open Access license.

## Electronic supplementary material

Supplementary information

## Data Availability

All data are fully available without restriction.
