# Supplementary material for: Functional recovery of a subtropical evergreen-deciduous broadleaved mixed forest following clear cutting in central China
Source: Sci Rep. 2018 Nov 7;8:16458. doi: 10.1038/s41598-018-34896-5 (PMC6220334; doi:10.1038/s41598-018-34896-5)
Supplement: Supplementary file 1 — Supplementary information [file 41598_2018_34896_MOESM1_ESM.docx]

**Functional recovery of a subtropical evergreen-deciduous broadleaved mixed forest following clear cutting in** **central China**

**Yongtao Huang^1,2^ Xiao Zhang^3^ Runguo Zang^2,4,*^ Shenglei Fu^3^ Xunru Ai^5^ Lan Yao^5^ Yi Ding^2,4^ Jihong Huang^2,4^ Xinghui Lu^2,4^**

^1^ *Post-Doctoral Research Program of Geography, College of Environment and Planning, Henan University, Kaifeng 475004, PR China*

^2^ *Key Laboratory of Forest Ecology and the Environment, the State Forestry Administration, Institute of Forest Ecology, Environment and Protection, Chinese Academy of Forestry, Beijing 100091, PR China*

^3^ *College of Environment and Planning, Henan University, Kaifeng 475004, PR China*

^4^ *Co-Innovation Center for Sustainable Forestry in Southern China, Nanjing Forestry University, Nanjing, Jiangsu 210000, PR China*

^5^ *School of Forestry and Horticulture, Hubei University for Nationalities, Enshi, Hubei 445000, PR China*

**Supplementary Figure**


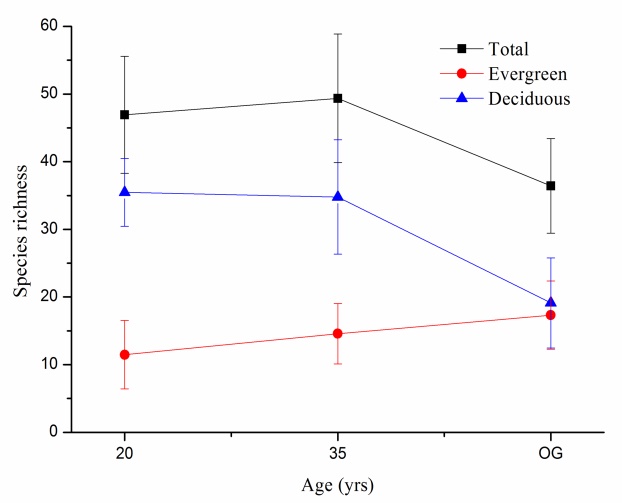

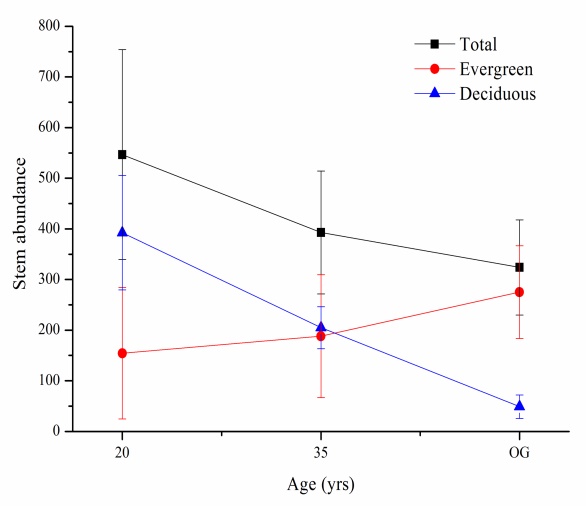


**Supplementary Figure S1.** Species richness and stem abundance in the subtropical evergreen-deciduous broadleaved mixed forest of different recovery stages. 20, 20-year-old second growth forest; 35, 35-year-old second growth forest; OG, old growth forest. Total, all species; Evergreen, evergreen species; Deciduous, deciduous species (Huang et al., 2015).


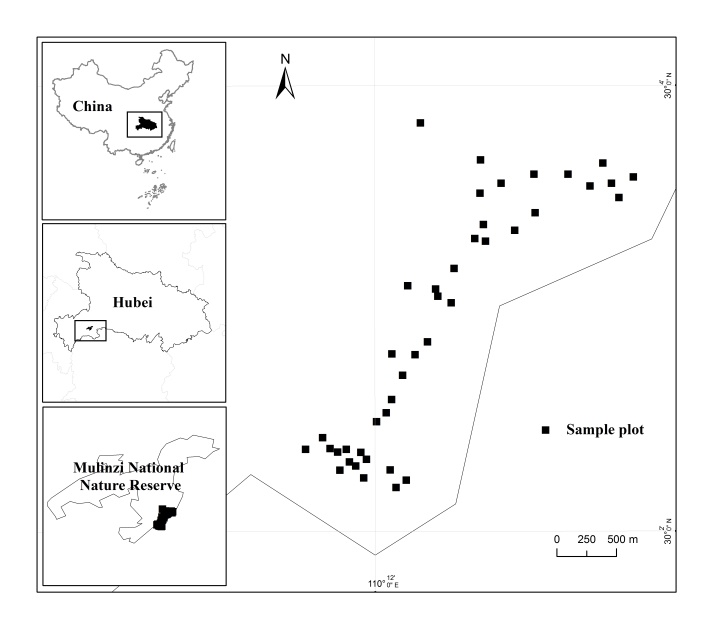


**Supplementary Figure S2.** Diagram of the sample plots and study site in southwest Hubei Province, central China (Huang et al., 2015).

**Supplementary Table**

**Supplementary** **Table S1** Step-wise multiple regression analysis between community-level plant fuctional trait values and stem abundance of deciduous species and evergreen species in the different recovery stages. 20SF, 20-year-old second growth forest; 35SF, 35-year-old second growth forest; OF, old growth forest. DSA, deciduous species abundance; ESA, evergreen species abundance. SLA, specific leaf area; LT, leaf thickness; STD, stem tissue density; LNC, leaf nitrogen concentration per mass; LPC, leaf phosphorus concentration per mass.

| Stage | Trait (CWM) | DSA | ESA | R^2^ | P < |
| --- | --- | --- | --- | --- | --- |
| 20SF |  |  |  |  |  |
|  | SLA | 0.42 | -0.18 | 0.55 | 0.01 |
|  | STD |  | 0.22 | 0.32 | 0.01 |
|  | LT | -0.09 |  | 0.21 | 0.05 |
|  | LNC |  | -0.14 | 0.18 | 0.05 |
|  | LPC | 0.33 | -0.29 | 0.27 | 0.01 |
| 35SF |  |  |  |  |  |
|  | SLA |  |  |  |  |
|  | STD |  | 0.17 | 0.19 | 0.05 |
|  | LT | -0.13 |  | 0.14 | 0.05 |
|  | LNC | 0.2 | -0.15 | 0.31 | 0.01 |
|  | LPC | 0.36 |  | 0.25 | 0.01 |
| OF |  |  |  |  |  |
|  | SLA | 0.28 | -0.24 | 0.41 | 0.01 |
|  | STD | -0.13 | 0.22 | 0.33 | 0.01 |
|  | LT |  | 0.16 | 0.17 | 0.05 |
|  | LNC | 0.29 |  | 0.25 | 0.01 |
|  | LPC | 0.18 |  | 0.16 | 0.05 |
